# Ca^2+^ Signaling and IL-8 Secretion in Human Testicular Peritubular Cells Involve the Cation Channel TRPV2

**DOI:** 10.3390/ijms19092829

**Published:** 2018-09-19

**Authors:** Katja Eubler, Carola Herrmann, Astrid Tiefenbacher, Frank-Michael Köhn, J. Ullrich Schwarzer, Lars Kunz, Artur Mayerhofer

**Affiliations:** 1 Biomedical Center Munich (BMC), Cell Biology, Anatomy III, Ludwig-Maximilian-University (LMU), D-82152 Planegg-Martinsried, Germany; katja.eubler@lrz.uni-muenchen.de (K.E.); carola.herrmann@lrz.uni-muenchen.de (C.H.); tiefenbacher@lrz.uni-muenchen.de (A.T.); 2 Andrologicum Munich, D-80331 Munich, Germany; info@andrologicum.com; 3 Andrology-Center-Munich, D-81241 Munich, Germany; J.U.Schwarzer@gmx.de; 4 Division of Neurobiology, Department of Biology II, Ludwig-Maximilian-University (LMU), D-82152 Planegg-Martinsried, Germany; lars.kunz@biologie.uni-muenchen.de

**Keywords:** Ca^2+^ signaling, interleukin-8, TRPV2, human testis

## Abstract

Peritubular cells are part of the wall of seminiferous tubules in the human testis and their contractile abilities are important for sperm transport. In addition, they have immunological roles. A proteomic analysis of isolated human testicular peritubular cells (HTPCs) revealed expression of the transient receptor potential channel subfamily V member 2 (TRPV2). This cation channel is linked to mechano-sensation and to immunological processes and inflammation in other organs. We verified expression of TRPV2 in peritubular cells in human sections by immunohistochemistry. It was also found in other testicular cells, including Sertoli cells and interstitial cells. In cultured HTPCs, application of cannabidiol (CBD), a known TRPV2 agonist, acutely induced a transient increase in intracellular Ca^2+^ levels. These Ca^2+^ transients could be blocked both by ruthenium red, an unspecific Ca^2+^ channel blocker, and tranilast (TRA), an antagonist of TRPV2, and were also abolished when extracellular Ca^2+^ was removed. Taken together this indicates functional TRPV2 channels in peritubular cells. When applied for 24 to 48 h, CBD induced expression of proinflammatory factors. In particular, mRNA and secreted protein levels of the proinflammatory chemokine interleukin-8 (IL-8/CXCL8) were elevated. Via its known roles as a major mediator of the inflammatory response and as an angiogenic factor, this chemokine may play a role in testicular physiology and pathology.

## 1. Introduction

Peritubular cells of human testis are cellular components of the wall of seminiferous tubules. They form several layers and, due to their contractile abilities, they are responsible for the transport of immotile sperm [1,2]. Besides these crucial functions for male fertility, newer studies imply that these cells possess a much broader spectrum of functions [3,4]. For instance, they are involved in paracrine signaling in the male gonad [5] and their participation in regulation of spermatogonial stem cells was shown for mice and men [6,7,8]. In addition, it became clear that in the human, peritubular cells can produce proinflammatory factors, express Toll-like receptors and have a say in the immune surveillance of the testis [9,10,11,12]. They may thus be players in sterile inflammation and male infertility [11,12].

A previous proteomic study of isolated human testicular peritubular cells (HTPCs) revealed, among others, expression of transient receptor potential cation channel subfamily V member 2 (TRPV2) [8]. While TRPV2 expression in organs such as brain, spleen and lung was described before [13], its expression in the testis is, to the best of our knowledge, not reported. 

TRPV2 is activated by mechanical stimuli [14,15], temperatures above 52 °C [16] or by chemicals such as 2-Aminoethyl diphenylborinate and cannabidiol [17,18]. Controversial results concerning activation and function are reported and may be due to species-related differences [19]. However, it is generally accepted that activation leads to an influx of cations and it has been shown, that channel permeability covers classical cations such as Ca^2+^ and Na^+^ with a ratio 2.9/1 [16,20]. Very recently the structural basis for permeation of not only metal ions but also large organic cations was provided [21,22]. Several studies reported that TRPV2 is involved in a wide range of physiological and pathological cellular processes. For instance, this non-selective cation channel was found to be pivotal for macrophage function [23], involved in innate and adaptive immune response [24], in promoting H_2_O_2_ cytotoxicity [25] and expression and secretion of cytokines [26].

The lack of information on TRPV2 in the human testis and its possible involvement in immune responses prompted us to study TRPV2 in human testis and in HTPCs.

## 2. Results

### 2.1. TRPV2 Expression in Cultured HTPCs and Human Testis

**Figure 1:**
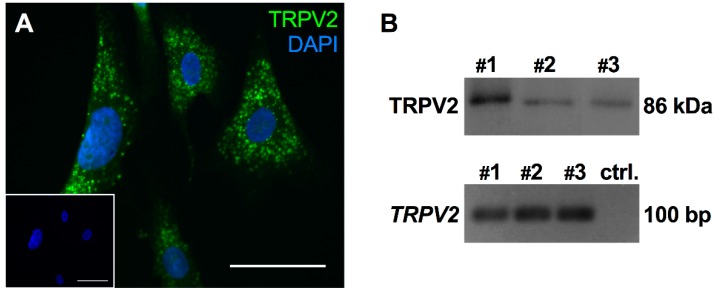
TRPV2 expression was confirmed in cultured HTPCs. (**A**) On the cellular level, TRPV2 exhibited a scattered expression pattern. Scale bar 10 µm. (**B**) In cultured HTPCs, expression of TRPV2 was detected by Western blot with a single band of the expected size (86 kDa) and RT-PCR showed clear bands at ~100 bp. Sequencing of the PCR-product validated expression of *TRPV2*. Bands obtained from three individual patients (#1–3) and negative control without cDNA in the RT-PCR reaction (ctrl.).

An immunocytochemical study confirmed the presence of TRPV2 in cultured HTPCs (Figure 1A). Using the same antibody for Western blotting, TRPV2 was readily detected in cultured HTPCs derived from three individual patients as a single band of the expected size (Figure 1B, upper panel). RT-PCR and sequencing further confirmed expression of *TRPV2* in cultured HTPCs (Figure 1B, lower panel).

**Figure 2:**
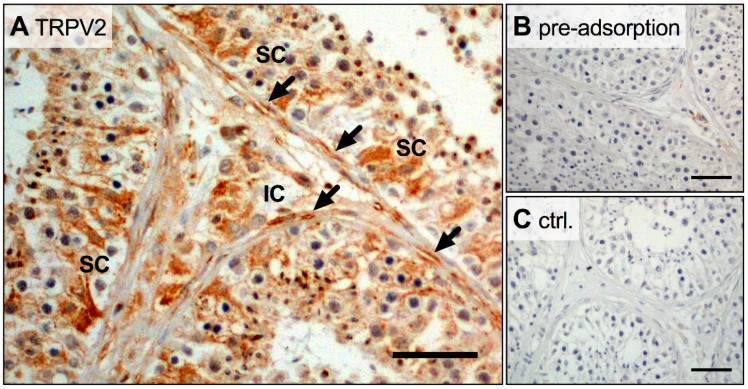
Broad expression of TRPV2 in human testis. (**A**–**C**) In human testicular slices, immunohistochemistry showed TRPV2 signals in Sertoli (SC), interstitial (IC) and in peritubular cells (arrows). In both controls, pre-adsorption with TRPV2 peptide (**B**) and omission of the primary antibody (**C**), no TRPV2 signal could be detected. Scale bar 100 µm.

In addition, immunohistochemical investigation of human testicular slices revealed expression of TRPV2 in several testicular cells, including peritubular cells, but also Sertoli and interstitial cells (including presumable Leydig cells) (Figure 2A). Control slices, where the TRPV2 antibody was pre-adsorbed or omitted, were negative (Figure 2B,C). These results indicate a widespread distribution of TRPV2 in the human testis. *TRPV2* expression levels in cultured HTPCs did not change when dihydrotestosterone (DHT; 10 µM) was added to the culture medium for 24 h or for 7 day (*n* = 5 and *n* = 3, respectively), implying that androgens do not play a role in the regulation of this channel.

### 2.2. Functionality of TRPV2 in HTPCs

**Figure 3:**
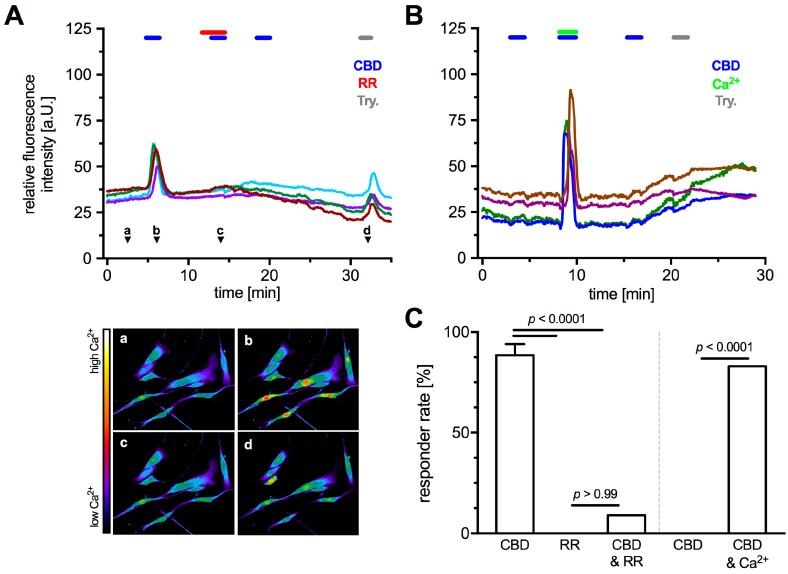
TRPV2 functionality as a Ca^2+^-channel in HTPCs was studied by applying the agonist CBD and the unspecific antagonists RR, in presence of CB1 and 2 blockers (AM251 and AM630), during Ca^2+^ imaging. (**A**) Application of TRPV2 activator CBD (10 µM) in presence of 1 mM extracellular Ca^2+^ led to a transient increase in intracellular Ca^2+^ that could be blocked completely by RR (10 µM). (**a**–**d**) Relative Ca^2+^ concentrations at the indicated time points (**A**, arrow heads) as pseudo color images (red = high Ca^2+^; purple = low Ca^2+^). (**B**) In absence of extracellular Ca^2+^ (0 mM), CBD alone did not induce any transients, whereas the combined application with Ca^2+^ (1 mM) resulted in Ca^2+^ transients, most likely due to Ca^2+^ influx. Each graph shows original traces of four representative cells from different donors (in total 5). In all experiments, trypsine (1‰) served as positive control. (**C**) The total rate of responding cells upon CBD application was 88.6 ± 5.5% (121 cells from 5 donors). No reaction occurred upon RR application (0%), but combined application with CBD led to transients in 9.1% of the analyzed cells (11 cells from 2 donors). In absence of any extracellular Ca^2+^, cells did not show reactions to CBD application (0%), whereas combined application with extracellular Ca^2+^ (1 mM) led to a responder rate of 83% (11 cells from 2 donors). The data were analyzed using Fisher’s exact test.

**Figure 4:**
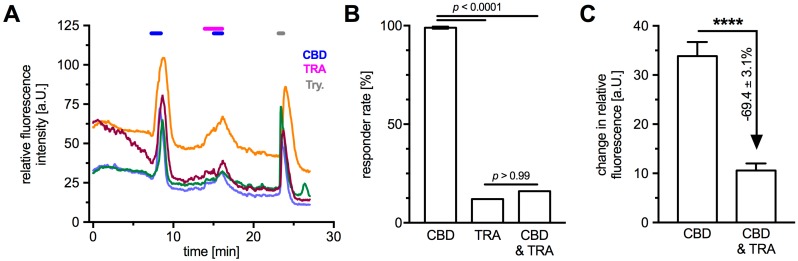
Application of the TRPV2-blocker TRA provided further evidence for functionality of TRPV2 in HTPCs during Ca^2+^ imaging. (**A**) In presence of CB1 and CB2 blockers (AM251 and AM630), application of TRPV2 activator CBD (10 µM) led to a transient increase in intracellular Ca^2+^ that could partially be blocked by TRA (10 µM). Graph shows original traces of four representative cells; three individual donors were analyzed. In all experiments, trypsine (1‰) served as positive control. (**B**) The total rate of responding cells upon CBD application was 99.0 ± 0.6% (25 cells from 3 donors). TRA application elicited transients in 12% of the analyzed cells and combination with CBD led to a responder rate of 16% (25 cells from 3 donors). The data were analyzed using Fisher’s exact test. (**C**) Application of TRA significantly reduced the measured changes in fluorescence intensity compared to the initial CBD-induced Ca^2+^ transients (**** *p* < 0.0001; 25 cells from 3 donors).

Being a non-selective cation channel permeable for Ca^2+^, functionality of TRPV2 in cultured HTPCs was examined by monitoring intracellular Ca^2+^ levels upon application of the known activator CBD and two blockers, ruthenium red (RR) and tranilast (TRA). Specificity of CBD to TRPV2 was ensured by the extracellular solution continuously containing the cannabinoid receptor 1 (CB1)-, and 2 (CB2)-blocker AM251 (80 nM) and AM630 (800 nM), respectively. With 1 mM Ca^2+^ in the extracellular solution (Figure 3A and Figure 4A), acute application of CBD (10 µM) led to a rapid and strong transient increase of intracellular Ca^2+^ level in most analyzed cells (responder rate 88.6 ± 5.5%, *n* = 121 cells from 5 patients). RR (10 µM), a non-selective Ca^2+^ channel blocker, did not affect intracellular Ca^2+^ levels (responder rate 0%, *n* = 11 cells), but blocked the CBD induced transients in nearly all cells (responder rate 9%, *n* = 11 cells; Fisher’s exact test, *p* < 0.0001; Figure 3A,C, left part). TRA (10 µM), recently described as TRPV2 blocker [27,28], reduced the number of responding cells (responder rate 16%, *n* = 25 cells) and led to a decrease in fluorescence change of 69.4 ± 3.1% compared to the initial CBD application (Figure 4). CBD-induced changes in intracellular Ca^2+^ levels were not seen when Ca^2+^ was omitted from the extracellular solution (responder rate 0%, *n* = 11 cells); however, the combined acute application of CBD and Ca^2+^ (1 mM) in this Ca^2+^ free environment elicited an increase of intracellular Ca^2+^ levels in 82% of the analyzed cells (*n* = 11 cells; Fisher’s exact test, *p* < 0.0001; Figure 3B,C (right part)), revealing an extracellular source of the transients.

Also, the striking nuclear Ca^2+^ influx upon CBD application (Figure 3A(b)) observed in all sets of experiment must be noted although it was not investigated in detail. Furthermore, application of CBD did not evoke any notable changes in cell size or shape.

### 2.3. TRPV2 Activation Induced Expression and Secretion of Cytokines in HTPCs

**Figure 5:**
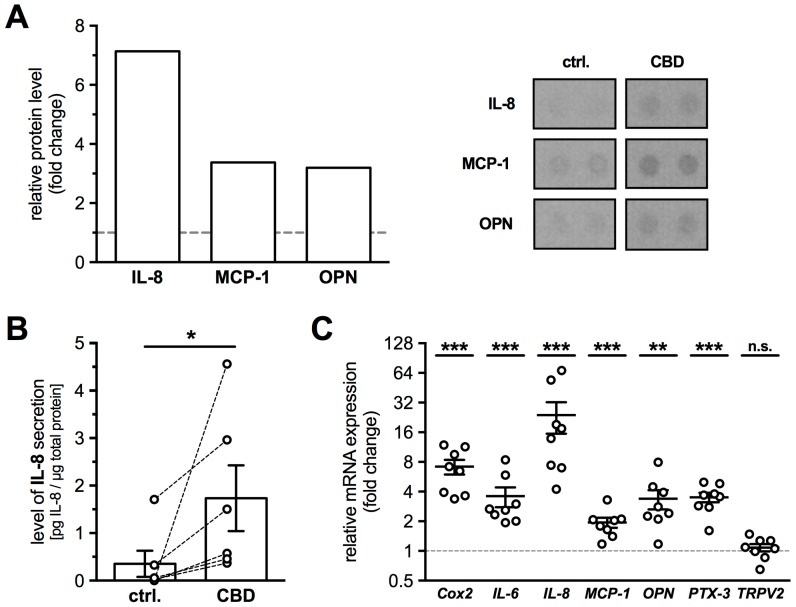
Activation of TRPV2 induced secretion of cytokines, especially of IL-8. (**A**) HTPCs preincubated with AM251 (80 nM) and AM630 (800 nM), and treated for 48 h with 10 µM CBD, exhibited increased secretion of IL-8 (7.1-fold), MCP-1 (3.4-fold) and OPN (3.2-fold) compared to untreated cells (*n* = 1), demonstrated by a Human Proteome Profiler. Right panel shows corresponding membrane spots of untreated control and CBD (10 µM) treated HTPCs revealing an increase of signal intensity of IL-8, MCP-1 and OPN. (**B**) Using an immunoassay, significantly increased IL-8 levels could be detected in culture media of HTPCs from six individual patients treated for 24 h with 10 µM CBD in the presence of CB1 and CB2 blockers (1.74 ± 0.69 pg/µg total protein), compared to the corresponding control condition (0.35 ± 0.28 pg/µg total protein). Results were normalized to total protein amount and statistically analyzed using Wilcoxon test (two-tailed, paired test: *p* = 0.0312; *n* = 6). (**C**) Quantitative PCR revealed significantly increased mRNA expression levels of *Cox2*, *IL-6*, *IL-8*, *MCP-1*, *OPN* and *PTX-3* in HTPCs after 24 h stimulation with 10 µM CBD and CB1 and 2 blockers. mRNA levels of *TRPV2* did not show any significant changes (1.08 ± 0.09-fold; *p* = 0.5905). *n* = 8; * *p* ≤ 0.05, ** *p* ≤ 0.01, *** *p* ≤ 0.001 vs. control cells.

Based on several publications, which linked TRPV2 to inflammatory processes, a screening experiment was performed employing a commercial cytokine proteome profiler assay. With CB1 and 2 blocked, CBD treatment (10 µM) for 48 h increased the medium levels of Interleukin-8 (IL-8; 7.1-fold), Monocyte Chemoattractant Protein-1 (MCP-1; 3.4-fold) and Osteopontin (OPN; 3.2-fold) (Figure 5A; *n* = 1).

In addition, several other factors were found to be slightly elevated in the culture medium (>2-fold change: Insulin-like growth factor-binding protein-3, Interleukin-17A, Urokinase receptor). As IL-8 secretion showed the strongest increase upon CBD treatment, HTPCs from 6 more patients were investigated using an immunoassay. CBD treatment (24 h, 10 µM) resulted in significantly increased IL-8 levels in the culture media (Figure 5B), with 0.35 ± 0.28 pg/µg in supernatants from untreated and 1.74 ± 0.69 pg/µg in those from treated cells (Wilcoxon test, *p* = 0.0312; *n* = 6). Also, at the mRNA level, expression of *IL-8* (23.91 ± 7.88-fold, *p* < 0.0001), *MCP-1* (1.94 ± 0.21-fold, *p* = 0.0008) and *OPN* (3.38 ± 0.70-fold, *p* = 0.0012) were significantly increased in CBD treated cells (*n* = 8). Additionally, the mRNA levels of *Cox2* (8.34 ± 1.78-fold, *p* < 0.0001), *IL-6* (3.62 ± 0.78-fold, *p* = 0.0005) and *PTX-3* (3.24 ± 0.42-fold, *p* = 0.0002) showed significant increases. However, *TRPV2* itself did not show any changes (1.08 ± 0.09-fold, *p* = 0.5905) in expression level upon 24 h activation (Figure 5C; *n* = 8).

**Figure 6:**
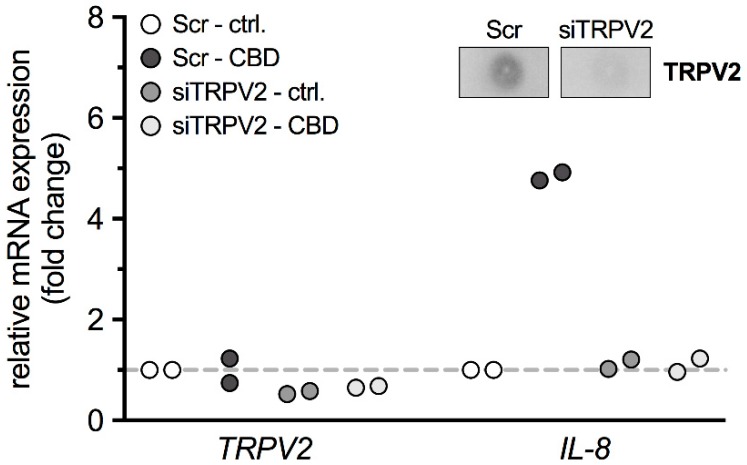
Transfection of HTPCs with a TRPV2 siRNA abolished the observed effects upon IL-8. Quantitative PCR data from HTPCs transfected with scrambled (Scr) non-targeting control siRNA or TRPV2 siRNA (siTRPV2) and treated with CBD (10 µM; Scr-CBD and siTRPV2-CBD) or equal volume of EtOH (Scr-ctrl. and siTRPV2-ctrl.) for 24 h. Upper panel shows a representative TRPV2 dot blot from HTPCs transfected with scrambled (Scr) non-targeting control siRNA and TRPV2 siRNA (siTRPV2), emphasizing the efficiency of the transfection.

To further test the specificity of CBD to activate TRPV2, cells from two donors were transfected with siRNA targeting TRPV2 or scrambled non-targeting control siRNA and then treated with CBD for 24 h (Figure 6). The transfection led to a reduction in TRPV2 protein amount (47.03 ± 16.94%; *n* = 2) and resulted in a slight decrease of *TRPV2* in cells transfected with the siRNA (siTRPV2-ctrl. 0.55 ± 0.03-fold, siTRPV2-CBD 0.67 ± 0.02-fold; *n* = 2 each), whereas the non-targeting control siRNA showed no effects on mRNA expression levels of *TRPV2* (Scr-CBD 0.99 ± 0.25-fold; *n* = 2). In control cells, CBD increased mRNA levels of *IL-8* (4.84 ± 0.08-fold; *n* = 2), whereas no changes in *IL-8* could be observed in the silenced cells (siTRPV2-ctrl. 1.11 ± 0.10-fold, siTRPV2-CBD 1.10 ± 0.14-fold; *n* = 2 each). 

## 3. Discussion

To our knowledge TRPV2 has not been described in human testis before, yet a proteome analysis of HTPCs had indicated its presence in this testicular cell type [8]. The present study confirms expression in HTPCs and their in situ counterparts, but also reveals an unexpected widespread expression of TRPV2 in the human testis. Results obtained in HTPCs implicate TRPV2 function in the release of IL-8.

Immunohistochemistry showed TRPV2 not only in peritubular cells but also in Sertoli cells, and interstitial cells, including presumably Leydig cells and immune cells of the human testis. We did not attempt to analyze their nature but rather focused on peritubular cells, because in contrast to other testicular cells, they can be isolated and studied in vitro.

Peritubular cells are smooth muscle-like cells and are thought to be pivotal for the transport of immotile sperm. Recent results obtained in HTPCs showed that they also contribute to the spermatogonial stem cell niche [6,29] and that they have immunological properties [11,12]. Peritubular cells possess androgen receptors, yet expression levels of *TRPV2* in these cells was not affected by DHT, a potent androgen, added to the culture medium for up to 7days. Such a treatment led to an increased expression of a growth factor (*PEDF*) in HTPCs [29], as well as smooth muscle markers and androgen receptors [30].

To investigate the role of TRPV2, the known pharmacological activator CBD was used [17]. Since at least CB1 expression was shown in HTPCs, CBD in combination with the CB1 and 2 blockers, AM251 and AM630, respectively, were employed as done before [18]. Application of CBD during Ca^2+^ imaging experiments resulted in transient increases of intracellular Ca^2+^ levels. Specificity of CBD, acting on TRPV2, was demonstrated by the application of the Ca^2+^ channel blocker RR and the TRPV2 antagonist TRA, resulting in significant reduction or even absence of any transients. Additionally, the external source of these transients was shown by omission of any transients during CBD application in absence of extracellular Ca^2+^. The measurements support functionally active TRPV2 in HTPCs.

Expression of TRPV2 is reported in dendritic cells, granulocytes, lymphocytes, monocytes, and macrophages [24,31], i.e., cells where the concentration of intracellular Ca^2+^ is crucial for their proper functionality. In HTPCs, we did not observe obvious changes in cell size or any other hints of contractions associated with increases of intracellular Ca^2+^ levels. Such changes in HTPCs were previously seen, for example, upon addition of angiotensin II [10]. Hence CBD-induced activation of TRPV2 may not be linked to the regulation of contractility of HTPCs. This point remains to be further investigated as it was suggested to occur in retinal arterioles [14].

Several reports have linked TRPV2 to inflammation e.g., in rheumatic diseases [32], experimental colitis [33] and oral inflammation [26], rather than to thermal sensation as described earlier [16]. Furthermore, TRPV2 was found to be enriched in several cancer types such as bladder and prostate cancer [34,35]. It was shown that activation of TRPV2 led to elevated levels of IL-6 and IL-8 in human periodontal ligament cells, and that deficiency of TRPV2 resulted in reduced macrophage infiltration and impaired phagocytosis [23]. As HTPCs are sources for cytokines [11,12], we explored consequences of TRPV2 activation. Despite expected heterogeneity between the samples stemming from individual patients [11,36], a proinflammatory influence of CBD became apparent with robustly increased levels of IL-8 in different experimental approaches. We focused on IL-8.

IL-8 is a known chemotactic and inflammatory cytokine being involved in recruitment of neutrophils, macrophages and mast cells into inflammatory sites and their activation [37,38,39]. The expression of the corresponding receptors for IL-8, i.e., CXCR1 and 2, was demonstrated also in human phagocytes, lymphocytes, and endothelial cells [40,41]. IL-8 has been reported in the male genital tract, especially in prostate and seminal plasma in health and disease [42,43]. In contrast, a role of IL-8 in normal testicular function remains to be determined [44] as testicular expression of the corresponding receptors for IL-8, CXCRs, was reported to be low or even absent. Yet, in cases of male infertility, which is associated with signs of sterile inflammation, it seems possible that IL-8 may have a say [11,12,45,46]. IL-8 is also involved in regulation of angiogenesis [47,48] with proangiogenic properties such as promoting proliferation, migration and tube-forming ability shown for human umbilical vein endothelial cells [49] and human aortic endothelial cells [50]. Promotion of proliferation and angiogenesis was also shown for colorectal cancer cells [51] revealing a link to cancer biology [52]. As the microvessel network contributes to the spermatogonial stem cell niche of the testis and because HTPCs produce pro- and anti-angiogenic factors [53], IL-8 may be a further factor involved.

In summary, our study describes testicular TRPV2 and identified a role in the regulation of proinflammatory factors, especially IL-8, in HTPCs. Beside peritubular cells, other testicular cells also possess TRPV2 and its specific involvement in the regulation of these cells is unknown. Furthermore, the physiological mechanisms leading to TRPV2 activation in the testis are not known. It is tempting to speculate that mechanical stimuli could be involved.

## 4. Materials and Methods

### 4.1. Isolation and Cell Culture of HTPCs

HTPCs stem from small samples of testicular tissue from vasectomized men from 36–55 years of age (*n* = 11) with obstructive azoospermia but normal spermatogenesis, as described [3,9]. The local Ethics Committee (Ethikkommission, Technische Universität München, Fakultät für Medizin, München, project number 5158/11, 18 October 2011; and project 309/14; 28 August 2014) has approved the study. The scientific use of the cells was permitted by written informed consent from all patients. The experiments were carried out in accordance with the relevant guidelines and regulations. Dulbecco’s Modified Eagle Medium high glucose (DMEM; Gibco, Paisley, UK) with 10% fetal calf serum (FCS; Capricorn Scientific, Ebsdorfergrund, Germany) and 1% penicillin/streptomycin (P/S; BioChrom, Berlin, Germany) was used for cell culture (37 °C, 5% CO_2_, 95% humidity). For experiments, HTPCs from passages 8–13 were used.

### 4.2. Immunohistochemistry and Immunofluorescence

Slices from human testicular sections of patients with normal spermatogenesis were used for immunohistochemical (IHC) and cultured HTPCs for immunocytochemical (ICC) staining as described before [3,54]. A polyclonal TRPV2 antibody (1:200; HPA044993, Atlas Antibodies, Stockholm, Sweden) was used for both methods. For IHC, a biotinylated α-rabbit secondary antibody (1:2500; Vector Laboratories, Inc., Burlingame, CA, USA), an avidin-biotin complex peroxidase (ABC, Vector Laboratories, Inc., Burlingame, CA, USA) and DAB (Sigma Aldrich, St. Louis, MO, USA) were used, followed by slight counterstaining with hematoxylin. For ICC, a fluorescence tagged secondary antibody (1:800; goat α-rabbit Alexa 488, life technologies, Carlsbad, CA, USA) and DAPI were used. Omission of primary antibody and pre-adsorption (1:100; APrEST83822, Atlas Antibodies, Stockholm, Sweden) served as negative controls.

### 4.3. RT-PCR and qPCR

Total RNA was extracted with the RNeasy Micro Kit (Qiagen, Hilden, Germany) and subjected to reverse transcription followed by real time PCR (LightCycler 96^®^ System, Roche Diagnostics, Penzberg, Germany) using the QuantiFast SYBR Green PCR Kit (Qiagen, Hilden, Germany) as described [10]. Changes in gene expression were normalized to the geometric mean of mRNA levels of *Peptidylprolyl isomerase A* (*PPIA*) and *ribosomal protein L19* (*L19*) as internal controls and analyzed according to the 2^−∆∆*C*q^ method, as described elsewhere [55]. Information on primers are given in Table 1. PCR products were visualized using a Midori Green Advance DNA stain (Nippon Genetics Europe, Düren, Germany) in a 2% agarose gel and sequenced to verify identity (GATC, Konstanz, Germany).

### 4.4. Immunoblotting

Immunoblotting was performed with HTPC whole cell lysates, as described elsewhere [56] employing the same antibody as used for IHC and ICC (polyclonal TRPV2 antibody; HPA044993, Atlas Antibodies, Stockholm, Sweden). Western blot or dot blot bands were detected using a corresponding HRP-conjugated secondary antibody and chemiluminescent solutions (SuperSignal^®^ West Femto Maximum Sensitivity Substrate; Thermo Fisher Scientific, Rockford, IL, USA).

### 4.5. Reagents

Dihydrotestosterone (DHT; Sigma Aldrich, St. Louis, MO, USA) was used to evaluate potential hormonal regulation of *TRPV2* expression. Channel functionality was examined by application of cannabidiol (CBD), as reported elsewhere [3]. CBD is known to also activate cannabinoid receptors and at least presence of cannabinoid receptor 1 (CB1) was detected. Therefore, experiments were carried out in presence of CB1 and CB2 blockers, AM251 and AM630, respectively [3]. In addition, the unspecific Ca^2+^ channel blocker ruthenium red (RR) and the recently described TRPV2 blocker tranilast (TRA) were used [13,57]. CBD, AM251, AM630 and RR were purchased from Tocris Bioscience (Bristol, UK), TRA was from Cayman (Ann Arbor, MI, USA). Stock solutions were dissolved in EtOH, DMSO or H_2_O, respectively. Application of equal concentrations of the corresponding solvent was used as control.

### 4.6. Ca^2+^ Imaging

For Ca^2+^ imaging experiments, HTPCs were loaded with the Ca^2+^ sensitive fluorescent dye Fluoforte^®^ Reagent (5 µM, Enzo Life Sciences, Lörrach, Germany) for 30 min at 37 °C and 5% CO_2_. Standard extracellular solution was composed of (in mM) 140 NaCl, 3 KCl, 10 HEPES, 10 glucose (pH 7.4 with NaOH), additionally AM251 (80 nM) and AM630 (800 nM) were added. The free extracellular Ca^2+^ concentration was set to 1 or 0 mM and that of CBD, RR and TRA to 10 µM. Trypsin (0.1 ‰, BioChrom, Berlin, Germany) served as a positive control at the end of every experiment. With excitation and emission wavelengths of 488 nm and 520 nm, fluorescence intensity was monitored using a confocal microscope (Axiovert 200M; Carl Zeiss Microscopy, Jena, Germany) equipped with a laser module (LSM 5, Carl Zeiss, Jena, Germany) and the software AIM 4.2 (Carl Zeiss MicroImaging, Jena, Germany). Data are expressed as relative fluorescence intensity based on a pseudo color scale from black/purple (low Ca^2+^) to white/red (high Ca^2+^) in arbitrary units (a.U.).

### 4.7. Treatment of Cells

For treatment, cells were serum starved 24 h prior to application of any drug. All stimulations were performed in FCS-free medium. For investigation of hormonal *TRPV2* regulation, cells were stimulated with DHT (10 µM) for 24 h or 7 d. For functional analysis of TRPV2, cells were preincubated for 1 h with AM251 (80 nM) and AM630 (800 nM) and then stimulated with CBD (10 µM) for 24 h for qPCR experiments and for 48 h for Human XL cytokine array.

### 4.8. Supernatant Protein Profiling

For the Proteome Profiler Human XL Cytokine Array (R&D Systems, Minneapolis, MN, USA), HTPCs were treated as described above and supernatants were collected. A total amount of 500 µL of supernatant was applied on the membranes according to the manufacturer’s instructions. Quantitative analysis was performed using Fiji software [58]. For each membrane, average spot signal density was determined by densitometry, followed by background subtraction and normalization to the respective protein concentrations.

### 4.9. IL-8 Immunoassay

Following the manufacturer’s instructions, the level of IL-8 in the supernatant after 24 h incubation with CBD (10 µM) was determined using an IL-8 ELISA (Human IL-8/NAP-1 Platinum ELISA; affymetrix eBioscience, Santa Clara, CA, USA). The lowest detectable IL-8 concentration was 2.0 pg/mL and the inter-assay coefficient of variance was less than 5.3%. Samples from 6 different patients were analyzed and the levels of IL-8 were expressed in pg/µg total protein amount.

### 4.10. siRNA Transfection

As both RR and TRA are not well suited for 24 h stimulation due to described side effects [59], cells were transfected with TRPV2 siRNA to block the observed effects. At ~80% confluency, cells were transfected for 6 h with Lipofectamine^®^ 2000 Transfection Reagent (Invitrogen, Carlsbad, CA, USA) dissolved in DMEM (Gibco Paisley, UK) according to the manufacturer’s protocol. A control non-silencing siRNA (negative control siRNA, 5 nM, #1022076; Qiagen, Hilden, Germany) and a TRPV2 siRNA (TRPV2 Silencer^®^ Pre-designed siRNA, 40 nM, #AM16708; life technologies, Carlsbad, CA, USA) were used. Cells were starved on DMEM containing 2% FCS and 1% P/S for further 18 h, preincubated with AM251 and AM630 for 1 h and then treated for 24 h with CBD and EtOH as solvent control, as described above. Efficiency of silencing was verified by a dot blot using 5 µg of total protein, the above listed TRPV2 antibody and ß-actin as housekeeping control (monoclonal β-actin antibody, 1:10,000; A5441, Sigma Aldrich, St. Louis, MO, USA). The experiment was performed twice with similar results.

### 4.11. Data Analysis and Statistics

Data analyses were performed using GraphPad Prism 7.0 (GraphPad Software Inc., San Diego, CA, USA). For statistical analysis of qPCR data and changes in fluorescence intensity, two-tailed one-sample t-test based on given Gaussian distribution (Shapiro-Wilk normality test) was used and the level of significance was set to 5%. For the responder rate, the Fisher’s exact test was used, and significance was set to 5%. Immunoassay data of control condition did not achieve normal distribution (Shapiro-Wilk normality test: *p* = 0.0009) and therefore data were analyzed using Wilcoxon test. Data are presented individually for each patient and, for qPCR and ELISA data, also as mean ± SEM.

## Figures and Tables

**Table 1:** Oligonucleotide primer sequences and corresponding amplicon size.

Gene	Reference ID	Nucleotide Sequence	Amplicon Size
*L-19*	NM_000981.3	5′-AGG CAC ATG GGC ATA GGT AA-3′ 5′-CCA TGA GAA TCC GCT TGT TT-3′	199 bp
*PPIA*	NM_021130.4	5′-AGA CAA GGT CCC AAA GAC-3′ 5′-ACC ACC CTG ACA CAT AAA-3′	118 bp
*Cox2*	NM_000963.3	5′-CTT ACC CAC TTC AAG GGA-3′ 5′-GCC ATA GTC AGC ATT GTA AG-3	132 bp
*IL-6*	NM_000600.4	5′-AAC CTG AAC CTT CCA AAG ATG G-3′ 5′-TCT GGC TTG TTC CTC ACT ACT-3′	159 bp
*IL-8*	NM_000584.3	5′-TCT TGG CAG CCT TCC TGA-3′ 5′-GAA TTC TCA GCC CTC TTC-3′	190 bp
*MCP-1*	NM_002982.3	5′-AGG TGA CTG GGG CAT TGA T-3′ 5′-GAA GTG ATG GGT ATC CGG TC-3′	109 bp
*OPN*	NM_001040058.1	5′-TTT TCA CTC CAG TTG TCC CC-3′ 5′-TAC TGG ATG TCA GGT CTG CG-3′	109 bp
*PTX-3*	NM_002852.3	5′-TAG TGT TTG TGG TGG GTG GA-3′ 5′-TGT GAG CCC TTC CTC TGA AT-3′	110 bp
*TRPV2*	NM_016113.4	5′-CCA GCA AGT ACC TCA CCG AC-3′ 5′-CAG GCA TTG ACT CCG TCC TT-3′	100 bp
